# Effects of concert music on cognitive, physiological, and psychological parameters in the elderly with dementia: a quasi-experimental study

**DOI:** 10.1590/1980-5764-DN-2021-0088

**Published:** 2022-04-29

**Authors:** Luana Aparecida da Rocha, Bianca Franceschini Siqueira, Caroliny Eduarda Grella, Aline Cristina Martins Gratão

**Affiliations:** 1 Universidade Federal de São Carlos, Laboratório de Avaliação e Intervenção em Gerontologia, Departamento de Enfermagem, São Carlos SP, Brazil.; 2 Universidade Federal de São Carlos, Laboratório de Avaliação e Intervenção em Gerontologia, Departamento de Gerontologia, São Carlos SP, Brazil.

**Keywords:** Aged, Dementia, Music, Alzheimer Disease, Idoso, Demência, Música, Doença de Alzheimer

## Abstract

Non-pharmacological interventions, such as the use of music, have been shown to be important potential means of controlling adverse symptoms and signs resulting from chronic diseases already present in elderly patients with dementia.

**Objectives:**

The objective of this study was to analyze the effects of concert music on cognitive and physiological parameters, and behavioral and psychological symptoms in institutionalized elderly people with dementia.

**Methods:**

A descriptive-exploratory, quantitative, quasi-experimental study was conducted with 14 elderly people. They were allocated in intervention group (IG) (n=7) with eight sessions of music listening, once a week, for 2 months, and control group (CG) (n=7) with the same procedure but without listening to the music. All participants were assessed by Neuropsychiatric Inventory Questionnaire (NPI-Q) and Addenbrooke’s Cognitive Examination – Revised (ACE-R) before and after the intervention. Blood pressure (BP) data were obtained; heart rate (HR) and coherence were obtained through Cardioemotion during sessions. The data were analyzed using Fisher’s exact test and Student’s *t*-test.

**Results:**

There was a predominance of female participants, who were widowed and diagnosed with Alzheimer’s disease (AD) in both groups. A statistically significant reduction was found in the mean of apathy reduction (p=0.038) and the total mean of NPI-Q severity (p=0.033) (paired Student’s *t*-test) in IG. No significant differences were found in mean level of the pre- and post-analysis variables in CG.

**Conclusions:**

Concert music had a positive effect on the behavior of institutionalized elderly. Stimuli and possibilities of improving the current behavioral conditions are observed.

## RESUMO.

Intervenções não farmacológicas, como o uso da música, têm-se mostrado importantes meios potenciais de controlar os sintomas e sinais adversos decorrentes das enfermidades crônicas já instaladas em idosos com demência.

**Objetivos:**

Analisar o efeito da música de concerto sobre a cognição, parâmetros fisiológicos e sintomas comportamentais e psicológicos em idosos com demência institucionalizados.

**Métodos:**

Estudo descritivo-exploratório, quantitativo, quase experimental, realizado com 14 idosos. Eles foram alocados em: Grupo Intervenção (GI) (n=7), com oito sessões de audição musical, uma vez por semana, durante dois meses; e Grupo Controle (GC) (n=7), com o mesmo procedimento, porém sem a audição da música. Todos os participantes foram avaliados pelo Neuropsychiatric Inventory Questionnaire (NPI-Q) e Addenbrooke’s Cognitive Examination – Revised (ACE-R) antes e depois do período da intervenção. Foram obtidos dados de pressão arterial, frequência e coerência cardíaca por meio do *cardioemotion* durante as sessões. Os dados foram analisados pelos testes Exato de Fischer e *t* de Student.

**Resultados:**

Em ambos os grupos houve predominância de participantes do sexo feminino, estado civil de viuvez e com diagnóstico de Alzheimer. Foi encontrada redução estatisticamente significativa na média do desgaste na apatia (p=0,038) e média total do NPI-Q gravidade (p=0,033) (teste *t* de Student pareado) no GI. Para o GC, não foram encontradas diferenças significativas no nível médio das variáveis na pré e pós-análise.

**Conclusões:**

A música de concerto teve efeitos positivos no comportamento dos idosos institucionalizados. Nota-se que, em geral, ela trouxe estímulos e possibilidades de melhoria das condições comportamentais atuais.

## INTRODUCTION

Concurrently with population aging, the prevalence of dementia syndromes also increases. Many studies reported rates between 4.2 and 7.2% (≥65 years), with age having a direct influence with average of 1.2% in the 65–69 age group and 39.9% in the 90–94 age group^1^. Dementia (major neurocognitive disorder) is a clinical syndrome that leads to deterioration of cognitive domains, behavioral changes, and functional loss^2^.

Alzheimer’s disease (AD) is the most common cause of dementia, followed by vascular dementia (VD) and the coexistence of both characterize mixed dementia (MD), among others. AD is a progressive and degenerative neurological disease that compromises behavioral and cognitive processes, leading to reduction of memory functions and visuospatial skills and independence and autonomy loss^3^.

Common dementia framework is the presence of behavioral and psychological symptoms. The terminology Behavioral and Psychological Symptoms of Dementia (BPSD) determines the set of signs and symptoms associated with disturbances of perception, mood, behavior, and thought content, such as delirium, hallucination, agitation or aggression, dysphoria, anxiety, euphoria, apathy, disinhibition, irritability or emotional lability, loss of motor function, nocturnal behavior, appetite, and dietary changes. These signs and symptoms are an important factor to patient suffering and distress of caregivers^3^.

As a consequence of supporting people with dementia, it has been increasing the number of elderlies on Long-Term Institutions for the Elderly (LTIE) as an alternative in providing essential care for maintaining life and being capable of providing quality of life^4^. These environments offer care assistance but generally almost do not offer stimuli to the elderly.

Therefore, pharmacological treatment consists of psychotropic drugs (e.g., antipsychotics, antidepressants, anticonvulsants, mood stabilizers, cholinesterase inhibitors, and memantine), which are the main options for the treatment of BPSD currently available^5^, but which sometimes are less effective for the control of the disease^6^, being of great relevance to search for alternative therapeutic measures to the already established medications. As a result, there has been an increase in research on non-pharmacological interventions to reduce the symptom burden for people with AD and their caregivers^7^. Among the interventions, there is the use of music to minimize symptoms related to dementia syndromes, as well as an improvement in quality of life^8^.

In general, an intervention using music can promote cognitive stimulation^9^. This is possible because musical memory networks are separated from traditional memory networks and are spared until the final stage of disease, activating a wide net in the brain, instead of only one “music area”^9^. In addition to decreasing the process of cognitive impairment, music intervention can stimulate motor skills, improve quality of life, and reduce problematic behavior associated with dementia^10^.

Music presents well-established psychological effects, including induction and mood and emotional changes. Some music types, such as meditative or concert music, reduce neurohormone markers of stress. In addition, music has heart rate (HR) and blood pressure (BP) effects. Relaxing music is capable of decreasing HR and BP, while fast rhythm music increases the signs^11^.

There is evidence that concert music interferes in some aspects of physiological variables due to a balance between sympathetic and parasympathetic system, in favor of parasympathetic system, through the possible involvement of limbic brain areas that would modulate hypothalamic-pituitary functions. These changes have an impact on induction and mood and emotional changes and can lower stress levels, providing relaxation^11^.

It is a recent academic-scientific investigation process, at a national and international level, related to music interventions being used as therapy in healthcare institutions, such as LTIE. As a result, it is believed that this study contributes to the advancement of gerontological intervention practices, based on clinical evidence, which will clarify important issues and is not yet sufficiently resolved in relation to cognitive stimulus, satisfaction, and well-being with the use of music in institutionalized elderly people with dementia. Thus, the general objective of this study was to analyze concert music effects on cognitive and physiological parameters, and psychological and behavioral symptoms in institutionalized elderly people with dementia.

## METHODS

It is a descriptive-exploratory, quantitative, quasi-experimental study developed from September to December 2018. It is understood that the quasi-experimental design was chosen because the sample was intentional and not probabilistic, in which the intervention group (IG) belonged to an LTIE (A) and the control group (CG) represented the residents of another LTIE (B). The CG was listed in this study for the possibility of comparing two similar groups, which were not randomized; however, similarities between them were guaranteed in demographic and health variables, except for outcome variable to the possibility of testing a cause-and-effect relation.

This study was realized in two private LTIE in the state of São Paulo. Both have elderly profile similarities: people with cognitive, behavioral, physical, and mental limitations, associated with neurological diseases and common diseases in old age, diagnosed by geriatricians associated with the institutions.

Therefore, elderly people aged above 60 years were included, LTIE A and LTIE B residents, with clinical diagnosis of dementia (varied etiologies), responsive to verbal commands. Exclusion criteria were as follows: other serious psychiatric disorders diagnosis, such as bipolar affective disorder, schizophrenia, and other psychoses, and having uncorrected possessing deficit that would make it impossible to hear music.

**Figure 1:**
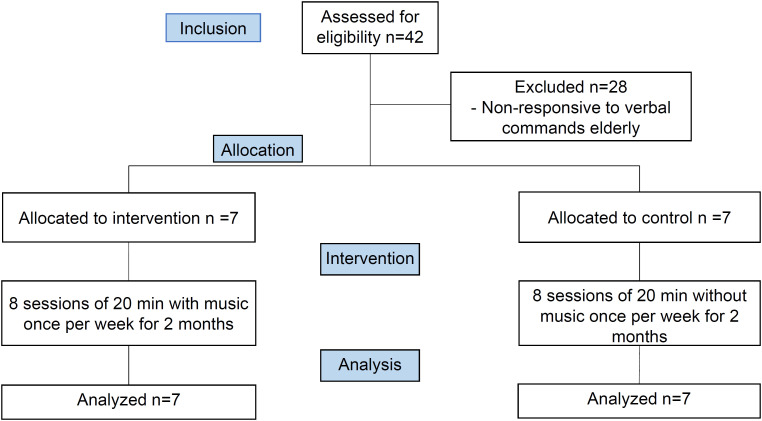
Participant allocation flowchart. São Carlos, SP, Brazil.

Notably, 14 elderly people participated in this research. Of a total of 42 individuals, 28 were excluded for not responding to verbal commands necessary for cognitive assessment. At LTIE A, 24 individuals were residents and 7 were assessed. At LTIE B, 18 individuals were residents and 7 were assessed (Figure 1).

The IG experienced one musical listening session of 20 min/week, consisting of concert music, for 2 months. The eight sessions always took place in the same appointments quiet room and at the same time (in the afternoon), in the institution itself, individually, realized by gerontologists. A headphone was placed in the auditory pavilion of the elderly, which was connected to a notebook for the complete emission of the music in a random sequence, in a 70-dB frequency. The songs were selected by a music therapists partner of the research group Laboratory of Evaluation and Intervention in Gerontology (LEIG), with the aim of causing relaxation, improving mood, and well-being, namely, Nocturne Opus 9 no. 2 by Frederic Chopin; Adagio in G minor (best live version) by Tomaso Albinoni; and Serenade for Winds (K. 361, 3^rd^ movement) by Wolfgang Amadeus Mozart. The criteria for selecting the songs were as follows: bars with a sense of continuity; harmony and repeated melodies with slower pace respecting the speed of cognitive processing of the elderly; and songs that rescued the collective unconscious and the lost sound identity (possibly hearing throughout life) and that were stimuli to maintain the attention of the elderly. In the CG, the participants went through the same process as the IG (headphone use), however without music, and were evaluated by the intervention protocol, and in the same way, they maintained their day-to-day activities at the institution.

The research protocol contained the systolic blood pressure (SBP) and diastolic blood pressure (DBP) verification before and after each musical hearing (was considered for analyzing the average of all sessions for each group). The HR and cardiac coherence (CC) were collected during the sessions and their values were obtained at the end of each session (for analysis, the data from the first and last sessions were considered), through biofeedback cardiovascular (Cardioemotion). The evaluation takes place by recording the time intervals, elapsed between each heartbeat, by an external sensor coupled to the second finger, followed by mathematical treatment of the data by the software^12^.

The nurses responsible for the two institutions (blind to intervention) were instructed to characterize the participants with demographic and health information and 1 month before and after the intervention, with the Neuropsychiatric Inventory Questionnaire (NPI-Q), a self-administered instrument, validated for evaluating the behavioral state of dementia patients during the last month^13^. This instrument assesses the severity (1–3) and the distress of caregivers (0–5) for 12 symptoms, namely, delirium, hallucination, agitation, depression, anxiety, euphoria, apathy, disinhibition, irritability, loss of motor function, nocturnal behavior, and dietary changes. The total NPI-Q score is obtained from the sum of the two subscales.

Addenbrooke’s Cognitive Examination-Revised (ACE-R) was used to assess cognition, also before and after the intervention period, by trained researchers from the research group. ACE-R evaluates six cognitive domains separately, namely, orientation, attention, memory, verbal fluency, speech, and visuospatial skill. The maximum score is 100 points, and the sum of all is equivalent to the individual’s total score in the ACE-R. Among this total, 30 relative points of the Mini-Mental State Examination (MMSE)^14^ are included.

The data were stored and processed in specific software for statistical analysis. The Shapiro-Wilk test was performed to assess data adherence to normality. The presence/absence of differences for continuous sociodemographic variables, at baseline, was verified using the independent samples Student’s *t*-test, and comparison of categorical variables using Fisher’s exact test. The presence/absence of differences between CG and IG of the outcome variables, before and after the intervention, was verified using Student’s *t*-test for paired samples. The level of significance adopted for all tests was p≤0.05 (5%).

The Research Ethics Committee of the Federal University of São Carlos approved this study, under opinion no. 1.981.699, in accordance with the provisions of Resolution no. 466/2012 of the National Health Council (NHC) and Resolution no. 510/2016, which defines the guidelines and regulatory standards that rule human being’s research. In addition, the informed consent form (ICF) was presented to the legal guardians of the participants and their consent was respected.

## RESULTS

In both groups, there was the predominance of widowed female participants diagnosed with AD. No statistically significant differences were identified between CG and IG in terms of demographic characteristics, with the exception of drugs, in which CG makes use of a greater number of medicines when compared to the IG (p=0.031). Table 1 shows the demographic variables of patients in each of the evaluated groups.

**Table 1:** Sociodemographic and health data of the intervention group and control group (n=14). São Carlos, SP, 2021.

Sociodemographic and health data		IG (n=7)	CG (n=7)	p-value
Gender [n(%)]	Male	0 (0.0)	2 (28.6)	0.462*
	Female	7 (100.0)	5 (71.4)	
Age [average (±SD)]		86.14 (±4.63)	81.29 (±7.99)	0.190**
Marital status [n(%)]	Married	1 (14.3)	0 (0.0)	0.378*
	Divorced/separated	0 (0.0)	1 (14.3)	
	Widowed	5 (71.4)	6 (85.7)	
	Single	1 (14.3)	0 (0.0)	
Education (years) [average (±SD)]		8.86 (±5.04)	6.86 (±5.42)	0.489**
Comorbidities (n)		2.57 (±0.97)	2.57 (±1.39)	1.000**
Medicines (n)		2.43 (±0.97)	5.43 (±3.10)	0.031**
Institutionalization time (months)		13.57 (±10.76)	6.71 (±5.93)	0.166**
Etiology of dementia [n(%)]	Alzheimer’s disease	7 (100.0)	3 (42.9)	0.061*
	Mixed dementia	0 (0.0)	2 (28.6)	
	Others	0 (0.0)	2 (28.6)	

IG: intervention group; CG: control group; n: number; SD: standard deviation; bold: statistically significant

* Fisher’s exact test

** Student’s *t*-test.

Table 2 shows the comparative data for the variables of severity and distress in NPI-Q, in addition to HR, SBP, DBP, CC, and ACE-R. It is worth to note the significant decrease in apathy reduction (*t*=2.646; p=0.038) and in the total mean of NPI-Q severity (*t*=2.760; p=0.033). Although not statistically significant, it is observed that some of the NPI-Q variables improved when compared to the moments before and after for IG, with lower averages in the post-analysis, such as the severity of depression and apathy, among others. For the CG, no significant differences were found in the mean level of the variables in the pre- and post-analysis, with the majority of the means remaining the same.

**Table 2:** Comparison of the pre- and post-intervention group (n=7) and the pre- and post-control group (n=7) for the outcome variables. São Carlos, SP, 2021.

	IG (n=7)			CG (n=7)		
	Pre-average (±SD)	Post-average (±SD)	*t* (p-value)	Pre-average (±SD)	Post-average (±SD)	*t* (p-value)
Delirium severity	1.57 (±0.97)	1.71 (±1.11)	-1.000 (0.356)	2.14 (±1.46)	2.14 (±1.46)	NR
Delirium distress	1.43 (±0.97)	1.29 (±0.95)	1.000 (0.356)	3.57 (±2.44)	3.14 (±2.41)	1.000 (0.356)
Hallucination severity	1.43 (±0.78)	1.29 (±0.75)	1.000 (0.356)	1.14 (±1.46)	1.14 (±1.46)	NR
Hallucination distress	1.57 (±0.97)	1.71 (±1.11)	-1.000 (0.356)	1.71 (±2.36)	1.71 (±2.36)	NR
Agitation severity	0.29 (±0.48)	0.29 (±0.48)	NR	0.71 (±1.25)	0.71 (±1.25)	NR
Agitation distress	0.29 (±0.75)	0.14 (±0.37)	1.000 (0.356)	1.00 (±1.91)	1.00 (±1.91)	NR
Depression severity	1.29 (±1.25)	0.86 (±0.90)	2.121 (0.078)	1.14 (±1.46)	1.14 (±1.46)	NR
Depression distress	1.43 (±1.51)	0.86 (±0.90)	1.922 (0.103)	1.71 (±2.36)	1.71 (±2.36)	NR
Anxiety severity	0.71 (±0.95)	0.57 (±0.78)	1.000 (0.356)	2.14 (±1.46)	2.14 (±1.46)	NR
Anxiety distress	0.71 (±1.11)	0.43 (±0.78)	1.549 (0.172)	3.57 (±2.44)	3.57 (±2.44)	NR
Motor disorder severity	1.00 (±1.29)	0.57 (±0.97)	1.000 (0.356)	0.43 (±1.13)	0.43 (±1.13)	NR
Motor disorder distress	1.29 (±1.70)	0.29 (±0.75)	1.732 (0.134)	0.71 (±1.89)	0.71 (±1.89)	NR
Nocturnal behavior severity	0.86 (±1.21)	0.43 (±0.53)	1.441 (0.200)	0.43 (±1.13)	0.43 (±1.13)	NR
Nocturnal behavior distress	0.71 (±1.25)	0.14 (±0.37)	1.333 (0.231)	0.71 (±1.89)	0.71 (±1.89)	NR
Appetite severity	0	0	NR	0	0	NR
Appetite distress	0	0	NR	0	0	NR
Euphoria severity	0.57 (±0.78)	0.43 (±0.53)	1.000 (0.356)	1.29 (±1.60)	0.86 (±1.46)	1.000 (0.356)
Euphoria distress	0	0	NR	1.86 (±2.41)	1.14 (±2.03)	1.000 (0.356)
Apathy severity	1.29 (±0.95)	0.86 (±1.06)	1.441 (0.200)	0.86 (±1.21)	1.00 (±1.29)	-1.000 (0.356)
Apathy distress	1.43 (±1.39)	0.43 (±0.79)	2.646 (0.038)*	1.57 (±2.14)	1.71 (±2.21)	-1.000 (0.356)
Disinhibition severity	0	0	NR	0	0	NR
Disinhibition distress	0	0	NR	0	0	NR
Irritability severity	0.29 (±0.75)	0.29 (0.75)	NR	1.29 (±1.60)	1.14 (±1.46)	1.000 (0.356)
Irritability distress	0.43 (±1.13)	0.43 (±1.13)	NR	2.14 (±2.67)	1.71 (±2.36)	1.000 (0.356)
NPI total severity	9.29 (±5.09)	7.29 (±4.23)	2.760 (0.033)*	11.57 (±6.13)	11.14 (±6.56)	0.701 (0.510)
NPI total distress	9.29 (±8.73)	5.71 (±6.18)	2.09 (0.081)	18.57 (±9.84)	17.14 (±11.29)	0.892 (0.407)
HR	71.14 (±5.61)	74.14 (±11.24)	-0.632 (0.540)	75.14 (±8.70)	73.71 (±12.93)	0.242 (0.813)
SBP average	121.57 (±6.52)	119.71 (±9.30)	0.432 (0.673)	126.43 (±6.90)	125.71 (±6.72)	0.196 (0.848)
DBP average	72.71 (±6.44)	70.29 (±6.77)	0.687 (0.505)	71.43 (±8.99)	68.57 (±7.48)	0.646 (0.530)
% coherence	24.57 (±8.16)	25.71 (±9.46)	-0.242 (0.813)	24.29 (±11.52)	24.43 (±8.05)	-0.027 (0.979)
MMSE	13.00 (±5.35)	13.29 (±4.19)	-0.111 (0.913)	14.00 (±6.75)	13.29 (±7.52)	0.187 (0.855)
ACE-R	37.29 (±13.42)	37.57 (±9.79)	-0.045 (0.964)	39.14 (±23.19)	36.29 (±21.89)	0.237 (0.817)
Attention/orientation	6.57 (±3.69)	6.57 (±3.10)	NA	7.29 (±4.07)	7.57 (±4.99)	-0.117 (0.909)
Memory	4.43 (±4.57)	4.43 (±3.10)	NA	8.57 (±5.38)	7.29 (±5.49)	0.442 (0.666)
Verbal Fluency	1.86 (±2.19)	1.57 (±2.44)	0.230 (0.822)	3.43 (±2.69)	2.71 (±3.03)	0.465 (0.650)
Speech	16.00 (±4.12)	16.57 (±4.23)	-0.256 (0.802)	12.71 (±7.74)	12.29 (±8.67)	0.098 (0.924)
Visuospatial skill	8.43 (±1.71)	8.43 (±1.39)	NA	7.14 (±5.01)	6.57 (±3.59)	0.245 (0.811)

* p<0.05 paired Student’s *t*-test; NR: not rated; bold: statistically significant.

## DISCUSSION

The elderly who participated in this study are long-lived, with an average age above 80 years, corroborating with other studies in this area, which evaluated institutionalized elderly people with dementia^15,16^. In addition, it is known that dementia is quite common in the elderly population, with a prevalence that doubles approximately every 5 years, starting at 65 years old^17^. There is also a predominance of women, which reflects on a feminization of old age, in which the elderly population majority, worldwide, is composed of women, due to a higher average of life expectancy when compared with men^18^. However, even if it seems positive, the high percentage of women in this study is associated with the high prevalence of dementia, in addition to the issue of widowhood and lack of support. The most prevalent type of dementia in the sample was AD, which is the most common, with an estimated number of 50 million people living with this disease in the world nowadays^19^.

This study shows that intervention with concert music has a positive impact on the behavioral symptoms of institutionalized elderly people with dementia and on the distress caused by them to the caregivers involved, mainly in the apathy symptom. Apathetic behavior is one of the neuropsychiatry symptoms most frequently reported in the elderly with dementia because it causes suffering for caregivers^3,20^. A study in China, with 77 institutionalized elderly people with dementia, evaluated the effect of a 12 week music therapy intervention on apathy. The IG received a musical intervention program, which included listening to nostalgic songs and playing instruments, e.g., xylophone. After 12 weeks of study, the apathy of patients undergoing the intervention showed a significant improvement, verified by Apathy Evaluation Scale (the pre- and post-intervention score difference, z=4.516, p<0.001), while the CG did not show a significant change in relation to this neuropsychiatric symptom. The cognitive function, evaluated by MMSE, was stable in the IG (t =1.720, p>0.05) but decreased in the CG (t =-1.973, p<0.05)^21^.

Concurrent with other studies, the result of this study suggested beneficial effects of the music intervention on the dementia symptoms and occupation disturbance, as measured by NPI^15,16,22^. In a study carried out in Spain with 42 institutionalized elderly with mild-to-moderate AD, who underwent music therapy for 6 weeks, a significant improvement was found in memory, orientation, depression, and anxiety (Hospital Anxiety and Depression Scale [HADS]) in patients with mild-to-moderate dementia; anxiety (NPI scale) in patients with mild dementia; and delirium, hallucinations, agitation, irritability, and language disorder in patients with moderate dementia. The effect on cognitive measures (MMSE) was noticeable after four music therapy sessions for all individuals^23^.

In another research conducted in Taiwan^24^, the authors selected concert music (Sonata by Mozart KV448 and Canon by Pachelbel), for the elderly to listen with headphones for 30 min daily in the morning and before bed, respectively, for 6 months. They found no behavioral alterations related to the intervention, but they found small cognition changes when examining the subcategories of cognitive tests (Cognitive Abilities Screening Instrument [CASI] and MMSE), contradicting the findings of this study, which did not obtain statistical differences in the cognitive domains of ACE-R.

In a study conducted in Australia, 99 institutionalized elderly participated in an experiment performed with a personalized playlist. The authors investigated the influence of depression, anxiety, apathy, and cognitive loss in the affective music response. Facial expressions were analyzed, and the behavioral responses were continuously observed. The results showed people with low depression, but high apathy levels demonstrated a greater behavioral evidence of pleasure during music listening. They concluded that music interventions are positive for people with dementia, but they need to consider the background and mental health symptoms of those involved^25^.

In a systematic review^26^, the authors suggested that the environments of long-term institutions contribute to the reduction of cognitive scores and that music is the path to improve patients’ quality of life. The concert music choice is still less used in research found in dementia literature context. Listening to music can work as a relaxation technique and, therefore, can have a long-term impact on the patients (symptom reduction), while active music therapy can set to involve the participants through social interaction and provide other benefits^7^. The findings suggest that musical techniques can be used in several ways to improve behavior and cognition^7^. Receptive music therapy can reduce restlessness, behavioral problems, and anxiety in the elderly with dementia and seems to be more effective than interactive music therapy in the treatment of dementia symptoms^6^.

Regarding the physiological parameters, no statistically significant differences were found, which may infer that the elderly remains stable, and hearing the concert music did not cause discomfort or altered physiological functions. A study conducted in Japan evaluated the effects of music therapy on the autonomic nervous system on plasma levels of cytokines and catecholamines in elderly with cerebrovascular disease and dementia, and in heart failure events. Notably, 87 elderly were evaluated in 10 sessions of 45 min with Japanese popular music. One study suggested that music therapy increased parasympathetic activities and decreased heart failure by reducing the plasma level of cytokines and catecholamines, providing well-being^27^.

Similar to other studies, this research is seeking a variety of promising discoveries related to dementia treatment. Pharmacological interventions are available but have limited capacity to treat many aspects of the syndrome^10^. Although most elderly people had the same diagnosis of dementia (AD), those in the CG used more medication (p<0.05) than the elderly in the IG, and it was not possible at this time to identify the class of drug used by each one, which did not allow for more in-depth analysis in these aspects.

The presence of BPSD is related to a greater cognitive impairment and the disease progression, although worsening the elderly life conditions and increasing the caregiver’s distress, as it requires a longer time of dedication and constant supervision by the professional^20^.

A limitation of this study is the reduced number of participants due to the difficulty of finding older adults with dementia who still respond to stimuli in the institutional environment, and the non-randomized sample may also have contributed to the lack of further findings. It is important to highlight that this study was not composed of music therapy sessions, but sessions using music, making up an elaborate intervention. In addition, the use of the NPI-Q by the nurse may have been insensitive, as this professional does not spend all the time with the patients due to shift changes; thus, the use of specific scales is suggested in future studies, such as the Apathy Scale and the Geriatric Depression Scale, applied directly to elderly participants.

Despite the limitations pointed out, the results showed that the intervention with concert music for institutionalized elderly people was effective in improving NIP severity symptoms and reducing stress on the professional in the apathy symptom in the IG. This study showed that stimuli and possibility to improve current behavioral conditions can be achieved and emphasizes the importance of continuing studies in this area. The result of well-designed interventions in the sociodemographic context can improve health care, safe, humanized, low cost, and easy-to-implement care. In addition, the relevance of identifying neuropsychiatric symptoms in the elderly with dementia diagnosis is perceived, so that health professionals can consider them in planning individualized care, as well as assisting caregivers for continuity of care with quality.
